# Electrochemical Oxidation Degradation of Methylene Blue Dye on 3D-Printed Anode Electrodes

**DOI:** 10.3390/polym17182499

**Published:** 2025-09-16

**Authors:** Claudia Cirillo, Mariagrazia Iuliano, Muhammad Shahzad, Emanuela Grazia Di Martino, Luca Gallucci, Nicola Funicello, Gerardo Iannone, Salvatore De Pasquale, Maria Sarno

**Affiliations:** 1 Department of Physics “E.R. Caianiello”, University of Salerno, Via Giovanni Paolo II, 84084 Fisciano, Italy; 2 NANO_MATES Research Centre, University of Salerno, Via Giovanni Paolo II, 84084 Fisciano, Italy; 3 National Institute for Nuclear Physics (INFN), P.le Aldo Moro, 2, 00185 Rome, Italy

**Keywords:** electrochemical oxidation, 3D-printed electrodes, methylene blue degradation, copper nanoparticles, wastewater treatment, conductive PLA composite

## Abstract

This study presents an innovative strategy for the electrochemical degradation of methylene blue (MB) using 3D-printed helical anode electrodes fabricated from commercially available conductive Polylactic acid/carbon black (PLA/CB) filaments. The choice of PLA/CB is particularly significant, since the amorphous PLA matrix combined with a percolating carbon black network provides a biodegradable, low-cost, and chemically versatile polymer composite that can be transformed from a simple prototyping filament into a functional electrochemical platform. Through a combination of chemical/electrochemical activation and electrodeposition of copper nanoparticles (Cu NPs), the polymer electrodes were successfully converted into highly efficient catalytic platforms. Beyond material functionalization, the influence of electrode geometry was systematically investigated, comparing single-, double-, and triple-spiral helical configurations. The double-spiral geometry proved the most effective, offering the best balance between active surface area and electrolyte flow dynamics. Under mild conditions (2 V, pH 6, 0.1 M NaCl), the system achieved up to 97% MB removal, while also demonstrating remarkable stability and reusability over at least ten consecutive cycles. These results highlight the synergistic role of polymer chemistry, arrangement, and metal decoration, demonstrating how 3D printing can be a useful platform for the easy production of electrodes with different geometries, even starting from simple conductive filaments reused in sustainable and scalable functional materials for advanced wastewater treatment.

## 1. Introduction

In recent years, increasing attention has been devoted to the treatment of organic pollutants such as methylene blue (MB) in industrial wastewater, particularly due to the rapid growth of the textile industry. This expansion has led to large volumes of dye-containing effluents, which are typically characterized by intense coloration and high chemical stability, representing a serious environmental concern. If discharged without adequate treatment, these effluents can cause severe ecological damage and pose risks to aquatic ecosystems and human health [1,2].

Among synthetic dyes, methylene blue (MB) is a heterocyclic aromatic compound with vivid coloration, high solubility, and a notable persistence in aqueous solutions. Its cationic nature and optical properties make it a representative model pollutant, widely used to evaluate the efficiency of novel wastewater treatment processes. Moreover, exposure to MB has been associated with adverse health effects, including skin irritation, permanent eye damage, and gastrointestinal disorders [3,4,5,6].

To address this issue, several treatment techniques have been investigated, including adsorption, chemical coagulation, ozonation, wet air oxidation, and electrochemical methods [4,7,8,9,10,11]. Among these, electrochemical oxidation has proven to be particularly effective, operating under ambient conditions and enabling the mineralization of organic pollutants into CO_2_ and water through the in situ generation of reactive oxygen species such as hydroxyl radicals at the anode surface [12,13]. The efficiency of this process, however, strongly depends on the electrode materials employed.

In this context, metallic nanoparticles have received considerable attention due to their ability to enhance charge transfer, increase mass transport at the electrode–solution interface, and improve catalytic activity [14,15,16,17]. At the same time, the development of additive manufacturing (AM) technologies, especially fused deposition modeling (FDM), has introduced new opportunities in electrochemistry. The use of 3D printing allows the fabrication of electrodes that are low-cost, customizable, and reproducible, with complex geometries and precise control over surface microstructures [18,19,20]. The most commonly available conductive thermoplastic filaments for 3D printing are PLA/graphene, PLA/carbon black (PLA/CB), PLA/Carbon Nanotubes (CNTs), and Acrylonitrile Butadiene Styren/CB (ABS/CB). A crucial aspect of their application is the formation of a percolating network of carbon nanomaterials within the polymer matrix. The electrical conductivity of these composites is governed by the so-called percolation threshold, i.e., the minimum concentration of nanofillers required to transform an insulating polymer into a conductive material. This threshold is strongly influenced by factors such as the type, shape, and size of the carbon fillers, their interactions with the polymer matrix, and the processing conditions, including dispersion and agglomeration [21]. Among these composites, PLA/CB is the most widely available and conductive. The amorphous PLA matrix offers biodegradability and printability, while the dispersed carbon black network ensures electrical conductivity. However, its application has been largely restricted to prototyping rather than functional use in environmental engineering. Critical aspects such as 3D geometry, surface activation, and post-functionalization have not yet been systematically studied [22,23]. Moreover, commercially available conductive filaments still face significant limitations, including low conductivity, small active surface area, sluggish charge-transfer kinetics, poor chemical stability, and limited compatibility with various electrolytes. These drawbacks are often attributed to intrinsic impurities and poor filler–matrix interactions. To overcome these challenges, several pre-activation treatments have been proposed to improve the electrochemical properties of conductive filaments and enable their use in high-performance electrodes [24]. For example, Browne et al. [25] demonstrated a combined chemical–electrochemical activation of graphene/PLA electrodes, consisting of immersion in DMF followed by the application of +2.5 V in phosphate-buffered saline, which enhanced charge-transfer kinetics and improved hydrogen evolution performance. Similarly, Santos et al. [26] applied a positive potential followed by voltammetric cycling, which improved charge transfer with [Ru(NH_3_)_6_]Cl_3_ and promoted the exposure of graphene sheets, enabling dopamine detection. Kalinke et al. [27] compared chemical activation (NaOH or *N*,*N*-dimethylformamide, DMF) with subsequent electrochemical activation, showing that electrochemical treatment alone already enhanced electron transfer, while the combination with NaOH proved most effective for dopamine determination. Beyond pre-activation, performance improvements have also been achieved through surface modification with metallic coatings or nanoparticles. As summarized in Table 1 [28,29,30,31,32,33,34,35,36,37], 3D-printed polymer electrodes decorated with metals such as Au, Ni, Cu, and Pt have been successfully employed in electrochemical applications ranging from the hydrogen evolution reaction to heavy metal and biomolecule sensing [38,39,40,41]. These modifications not only enhance conductivity and catalytic activity but also exploit the design flexibility of 3D printing for tailored electrode architectures.

Among different approaches, electrodeposition has emerged as a particularly attractive strategy, as it enables the direct growth of nanostructured metallic layers on polymer substrates in a simple and cost-effective manner [42,43,44].

In this study, the degradation of methylene blue (MB) was investigated through electrochemical oxidation using 3D-printed polymer-based electrodes. The anodes were fabricated via additive manufacturing (AM) employing a spiral-shaped geometry with a two-loop design and using a PLA/carbon black (PLA/CB) filament. This composite is of particular relevance to polymer science, as the amorphous PLA matrix combined with a percolating carbon black network enables the transition from an insulating thermoplastic to a conductive material suitable for electrochemical applications. The anode was subsequently activated and modified through copper electrodeposition, while a platinum electrode served as the cathode. The functionalized 3D-printed electrodes were characterized by cyclic voltammetry (CV) and electrochemical impedance spectroscopy (EIS) to assess their performance.

Operational parameters such as sodium chloride (NaCl) concentration, applied potential, pH, and electrode reusability were systematically investigated to optimize the electrochemical pretreatment and degradation efficiency of MB, used here as a model dye. Unlike traditional studies mainly focused on novel material synthesis, our work emphasizes how a commercially available conductive polymer filament—typically restricted to basic prototyping—can be repurposed into a high-performance electrochemical platform through scalable surface functionalization. By integrating the intrinsic properties of the PLA/CB polymer composite, copper nanoparticle electrodeposition, and innovative electrode geometry, this study highlights the synergistic role of polymer chemistry, filler distribution, and 3D architecture in driving electrocatalytic activity.

Furthermore, electrode geometry was extended beyond the basic configuration by comparing helical structures with different numbers of spirals. This comparison revealed their effectiveness in color removal and underlined the importance of three-dimensional polymeric structures in enhancing the active surface area and promoting electrolyte flow dynamics. To our knowledge, this is the first demonstration of the combined effect of electrode geometry and Cu nanoparticle functionalization on the electrochemical mineralization of methylene blue using fully 3D-printed polymer-based substrates.

## 2. Materials and Methods

### 2.1. Material

Protopasta (Vancouver, WA, USA) provided a commercial carbon black/polylactic acid (CB/PLA) filament for 3D printing (Table 2). All chemicals were used as received from Sigma-Aldrich (St. Louis, MO, USA) without any further purification. These included sodium chloride (NaCl, analytical grade), sulfuric acid (H_2_SO_4_, 98%), *N*,*N*-dimethylformamide (DMF), acetone, copper(II) chloride, copper sulfate, boric acid (H_3_BO_3_), and methylene blue (MB).

### 2.2. Electrode Fabrication

Conductive electrodes were fabricated using polylactic acid (PLA) via fused deposition modeling (FDM) with an Ultimaker 3S^®^ 3D printer (Ultimaker, Geldermalsen, The Netherlands). A commercially available conductive PLA filament from Protopasta served as the base material. The electrode geometry was designed using Tinkercad^®^ software (Autodesk, San Francisco, CA, USA), while the corresponding g-code files were generated using Ultimaker Cura^®^ slicer software (Version 5.2.2, Ultimaker, Geldermalsen, The Netherlands). Among various possible configurations, a double-helical geometry was selected due to its simplicity and suitability for additive manufacturing. This design enables enhanced contact between the electrode and the electrolyte, facilitates vertical flow dynamics, and promotes rapid gas bubble detachment from the surface, which are critical factors in preventing surface passivation and maintaining catalytic efficiency [31]. The total length of each electrode was 5 cm, comprising two helical turns. Optimized FDM printing parameters were as follows: nozzle temperature 200 °C, build plate temperature 50 °C, print speed 80 mm/s, first layer height 0.15 mm, layer height 0.1 mm, two perimeters per layer, 100% infill, 5 mm retraction distance at 40 mm/s retraction speed, 100% cooling fan speed, and a 0.4 mm nozzle diameter. The conductive printed electrode (referred to as C-3DPE) underwent a chemical activation treatment to enhance its electrical conductivity. This involved immersion in 5 mL of DMF for 24 h, following procedures reported in the literature [38,44,45]. The treatment promoted partial removal of the PLA matrix from the surface, thereby exposing the conductive filler. After chemical treatment, the electrode was thoroughly rinsed with ethanol and acetone to eliminate residual polymer and subsequently dried in a vacuum oven at 60 °C for 60 min. Final activation was carried out electrochemically in 0.1 M phosphate-buffered saline (PBS) by applying a constant potential of 2.5 V for 300 s using chronoamperometry with an Autolab PGSTAT302N potentiostat/galvanostat (Metrohm, Herisau, Switzerland). The fully treated electrode obtained through this protocol is referred to as AC-3DPE. 

### 2.3. Electrodeposition of Copper Nanoparticles (Cu NPs) on AC-3DPE

The electrodeposition of copper nanoparticles (Cu NPs) was carried out using a two-electrode chronoamperometric technique at a constant potential of 10 V for 1200 s [40]. The process was performed in an electrolyte solution containing 0.188 M CuSO_4_, 0.0074 M CuCl_2_, and 0.0202 M H_3_BO_3_ [46]. The electrode obtained from this deposition procedure is referred to as Cu@AC-3DPE.

### 2.4. Characterization of Modified Elecrodes

C-3DPE, AC-3DPE, and Cu@AC-3DPE samples were characterized using scanning electron microscopy (SEM) (TESCAN-VEGA LMH; 230 V; Tescan, Brno, Czech Republic). FT-IR spectra were obtained using a Nicolet iS50 FT-IR spectrometer (Nicolet, Waltham, MA, USA). X-ray diffraction measurements were also performed by a Bruker D2 diffractometer equipped using CuKα radiation (Bruker, Billerica, MA, USA). The electrodes at different stages of the sensor preparation were analyzed using X-ray diffraction (XRD) with a Bruker D2 X-ray diffractometer and CuKα radiation (Bruker, Billerica, MA, USA).

### 2.5. Electrochemical Degradation of MB

The methylene blue (MB) degradation experiments were conducted in a three-electrode electrochemical cell operating under ambient temperature and pressure. The system consisted of a Cu@AC-3DPE working electrode, a platinum counter electrode, and a Ag/AgCl (3 M KCl) reference electrode. The electrodes were vertically immersed in 100 mL of an aqueous electrolyte solution containing 50 mg/L of MB and a defined concentration of NaCl. The initial pH of the solution was adjusted to 6 using diluted HCl or NaOH, as needed. Continuous magnetic stirring at 300 rpm ensured homogeneous mixing of the solution throughout the experiment. A constant potential was applied to the working electrode using a potentiostat/galvanostat, and the resulting current was monitored versus the reference electrode. MB degradation was evaluated using UV-Vis spectrophotometry (Evolution™ 60S, Thermo Scientific™, Waltham, MA, USA), by monitoring the decrease in absorbance at 664 nm within the spectral range of 500–800 nm. The degradation efficiency of MB was determined using the following equation (Equation (1)):

(1) $$Electrochemical\;degradation\;\left(\%\right)=\frac{A_{0}-A_{t}}{A_{0}}\times 100$$

where *A*_0_ and *A_t_* represent the absorbance of the initial dye solution at the maximum absorption wavelength (λ_max_) and the absorbance at time *t*, respectively. The effect of NaCl concentration on the electrochemical degradation process was assessed at a fixed potential of 2 V, with NaCl concentrations set at 0.05, 0.1, and 0.2 M. These values are lower than the typical salinity of seawater (≈35 g/L), allowing for the evaluation of the process under low-salinity conditions. All experimental data were collected in triplicate and are reported as mean values ± standard deviation. Process parameters were optimized by varying the initial pH (3–9) and applied potential (0.5–2 V). Finally, the reusability of the prepared Cu@AC-3DPE anode was investigated. After each cycle, the electrode was regenerated by washing it three times with ethanol and bidistilled water, followed by drying at 110 °C.

### 2.6. Analysis of Chemical Oxygen Demand

Chemical Oxygen Demand (COD, mg O_2_/L) was determined following ISO 15705:2002 [47]. Briefly, 2 mL of each sample was collected in sealed glass vials before and after electrochemical degradation. Each vial received 4 mL of reagent, then was digested at 150 °C for 2 h using a COD reactor (VELP Scientifica, Usmate (MB), Italy). After cooling for 30 min, COD was measured with a COD meter.

To reduce interference from chlorides and electrogenerated species, especially at concentrations >2000 mg/L, sodium bisulfite (Na_2_S_2_O_5_) was added to eliminate residual hypochlorite and chlorine gas before analysis [32,48]. *COD* removal efficiency was calculated using Equation (2):

(2) $$COD\;Removal\left(\%\right)=\frac{{COD}_{0}-{COD}_{t}}{{COD}_{0}}\times 100$$

where *COD*_0_ and *CODₜ* represent the *COD* values before and after electrochemical degradation treatment, respectively.

## 3. Results and Discussion

### 3.1. Stepwise Fabrication of Cu-Modified 3D-Printed Electrodes

**Figure 1:**
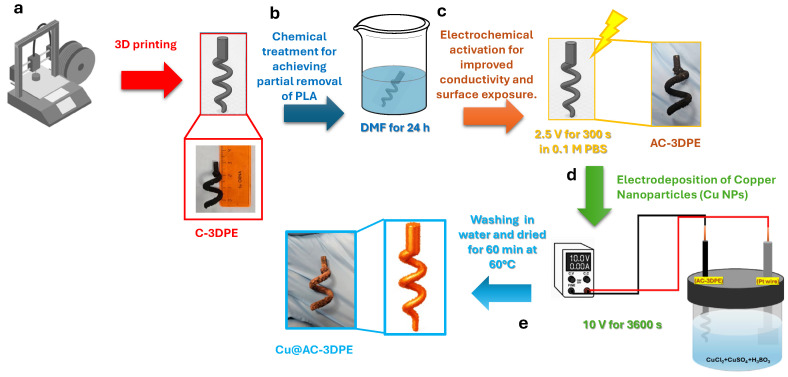
Schematic representation of the electrode fabrication process, involving the electrochemical deposition of copper nanoparticles (Cu NPs) onto a 3D-printed CB/PLA electrode with a double-helix architecture. The process consists of (**a**) 3D printing of the electrode; (**b**) chemical purification using DMF; (**c**) electrochemical activation; (**d**) electrodeposition of Cu NPs; and (**e**) washing and drying.

Figure 1 illustrates the electrode fabrication process. Conductive electrodes were fabricated via fused deposition modeling (FDM) 3D printing using a CB/PLA filament and a double-helix geometry (Figure 1a). Following printing, the electrode (referred to as C-3DPE) underwent chemical purification by immersion in DMF for 24 h to partially remove the polymeric matrix and enhance the exposure of the conductive filler (Figure 1b). The electrode was then rinsed with ethanol and acetone, followed by drying in an oven at 60 °C. Electrochemical activation was subsequently performed by applying a potential of 2.5 V for 300 s in 0.1 M PBS, yielding the activated electrode AC-3DPE (Figure 1c). Copper nanoparticles (Cu NPs) were then deposited onto the electrode surface via chronoamperometry at 10 V for 1200 s in an electrolyte solution containing CuSO_4_, CuCl_2_, and H_3_BO_3_ (Figure 1d). Finally, the electrode was rinsed, dried at 60 °C, and designated as Cu@AC-3DPE (Figure 1e).

### 3.2. Characterization of Cu@AC-3DPE

**Figure 2:**
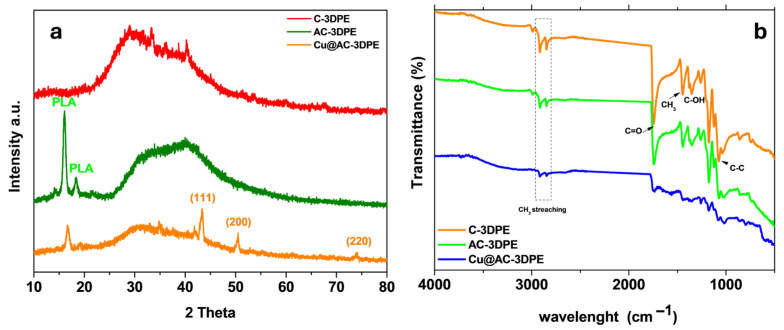
(**a**) XRD patterns of the C-3DPE (red line), AC-3DPE (olive line), and Cu@AC-3DPE (orange line) electrodes; (**b**) FT-IR spectra of the C-3DPE (orange line), AC-3DPE (green line), and Cu@AC-3DPE (blue line) electrodes recorded in the range of 4000–500 cm^−1^.

The structural properties of the fabricated electrodes were initially investigated using X-ray diffraction (XRD), as illustrated in Figure 2. The XRD pattern of the 3D-printed activated carbon electrode (AC-3DPE) displays distinct peaks at 16.17° and 18.42°, which correspond to the characteristic crystallographic planes of polylactic acid (PLA), as reported by Zhu et al. (2023) and Chen et al. (2011) [23,49]. The XRD spectrum of the Cu@AC-3DPE electrode is shown in Figure 2 (orange profile). This pattern exhibits characteristic diffraction peaks of Cu NPs corresponding to the (111), (200), and (220) planes of the face-centered cubic (fcc) crystalline structure [50], located at 2θ values of 43.5°, 50.5°, and 74.3°, respectively, in agreement with previous reports [51,52].

The crystallite size of the Cu NPs was estimated using the Scherrer equation (Equation (3)) [53]:

(3) $$D=\frac{K\lambda }{\beta \cos\theta }$$

where *K* is the shape factor, *λ* is the X-ray wavelength, *β* is the full width at half maximum (FWHM) of the diffraction peak (in radians), and *θ* is the Bragg angle. Based on this analysis, the average crystallite size of the copper nanoparticles was calculated to be approximately 10 ± 1.81 nm, indicating the successful formation of nanostructured Cu domains. Figure 2b presents the FT-IR spectra of the carbon black and PLA-based electrode (C-3DPE) after 3D printing (orange line), after activation (green line), and following the electrochemical deposition of copper nanoparticles (Cu@AC-3DPE, blue line). In the spectrum of the unmodified and activated electrodes, characteristic bands of PLA are observed, including the symmetric and asymmetric stretching vibrations of the methyl (CH_3_) group at 2919 cm^−1^ and 2846 cm^−1^, respectively. Also evident are signals associated with oxygen-containing functional groups of PLA, such as the carbonyl (C=O) stretching at 1738 cm^−1^ and C–O–H vibrations at 1347 cm^−1^. Additional bands at 1448 cm^−1^ and 1042 cm^−1^ are attributed to CH_3_ bending and C–C stretching, respectively [54,55,56,57]. The intimate interaction between the functional groups of PLA and the carbon structures of carbon black may lead to overlapping, shifting, and broadening of the bands, making it difficult to assign spectral contributions to individual components distinctly. After the deposition of Cu NPs, slight changes in the spectral profiles and a reduction in band intensities are observed, indicating a chemical–physical interaction between the metal nanoparticles and the CB/PLA matrix. Although subtle, these modifications support the occurrence of surface functionalization of the electrode, confirming the integration of the active material into the conductive composite structure.

**Figure 3:**
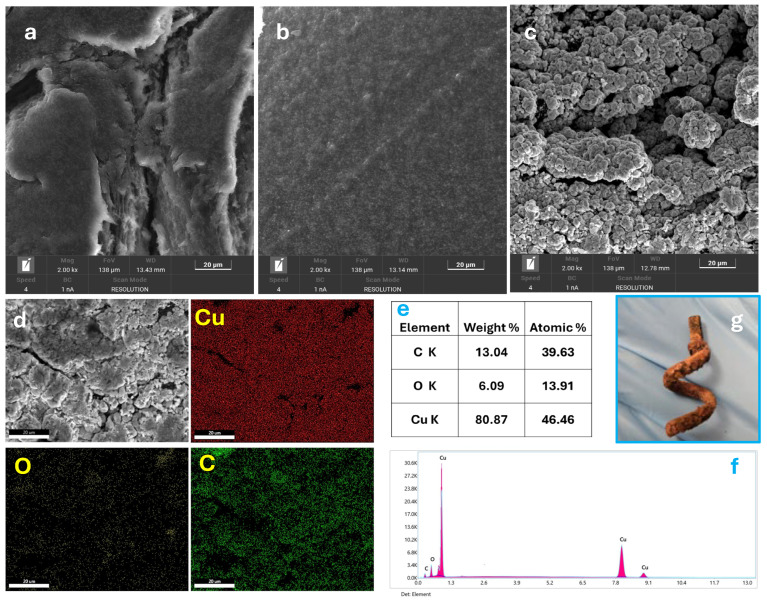
SEM images of the (**a**) C-3DPE, (**b**) AC-3DPE, and (**c**) Cu@AC-3DPE electrodes at a magnification of 2.00 kx; (**d**) SEM image and corresponding EDX elemental mapping of Cu@AC-3DPE, highlighting the distribution of elements; (**e**,**f**) composition and EDX spectrum of Cu@AC-3DPE; and (**g**) photograph of the Cu@AC-3DPE electrode (after Cu NP electrodeposition).

The surface morphology of the electrodes was investigated by scanning electron microscopy (SEM), as shown in Figure 3a–c. In Figure 3a, the surface of the as-printed C-3DPE electrode appears smooth and featureless due to the presence of the outer PLA layer, which, being non-conductive, masks the underlying carbon structures. After chemical treatment with DMF followed by electrochemical activation in 0.1 M PBS at 2.5 V for 300 s, the electrode surface undergoes substantial morphological changes (Figure 3b). The surface becomes more irregular and porous, revealing the underlying conductive carbon black particles, thus forming the activated electrode (AC-3DPE). Subsequent electrodeposition of Cu NPs onto the activated surface (Cu@AC-3DPE) results in the appearance of bright Cu nanoscaled aggregates dispersed across the surface, as visible in the SEM micrographs (Figure 3c). Elemental mapping (Figure 3d) confirms the spatial distribution of Cu, C, and O across the electrode, evidencing that copper is homogeneously integrated within the electrode surface. Energy-dispersive X-ray spectroscopy (EDX) analysis (Figure 3e,f) further supports these findings, showing a surface composition dominated by copper (46.46 at%) and carbon (39.63 at%), along with a measurable amount of oxygen (13.91 at%). The photograph in Figure 3g shows the Cu@AC-3DPE electrode featuring a three-dimensional helical structure, visibly coated with a porous surface layer of deposited Cu NPs onto the 3D-printed scaffold.

### 3.3. Electrochemical Characteristics

**Figure 4:**
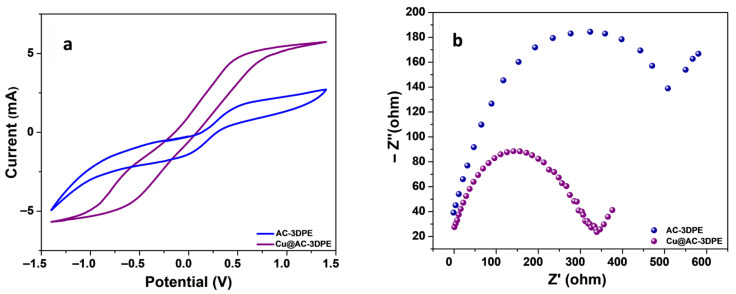
CV behavior of the AC-3DPE and Cu@AC-3DPE electrodes measured in the presence of 10.0 mM [Fe(CN)_6_]^3−/4−^ in 0.1 M KCl at 50 mV/s (**a**); and EIS behavior of the AC-3DPE and Cu@AC-3DPE electrodes measured in the frequency region from 50 kHz to 0.01 Hz at an AC potential ± 5 mV (**b**).

Figure 4a displays the CV curves of the AC-3DPE and Cu@AC-3DPE electrodes, following surface activation and subsequent copper electrodeposition. The AC-3DPE exhibits distinct redox peaks, which can be attributed to the exposure of conductive carbon black particles that were previously insulated by the PLA coating. After the electrodeposition of metallic copper onto the AC-3DPE surface, the resulting Cu@AC-3DPE electrode demonstrated a further enhancement in electrochemical activity. Specifically, the peak current increased by approximately 200% compared to the activated electrode, due to the improved conductivity and presence of the metallic copper layer. Electrochemical impedance spectroscopy (EIS), shown in Figure 4b, was employed to evaluate the charge-transfer resistance (Rct) of the electrodes. After activation, the AC-3DPE electrode exhibited an Rct of 515 Ω, significantly lower than that of the unmodified 3DPE, due to the removal of the non-conductive PLA layer. The subsequent copper modification further reduced the Rct to 345 Ω, indicating a substantial improvement in the electrode’s electronic conductivity.

#### 3.3.1. Electrochemical Oxidation of MB

**Figure 5:**
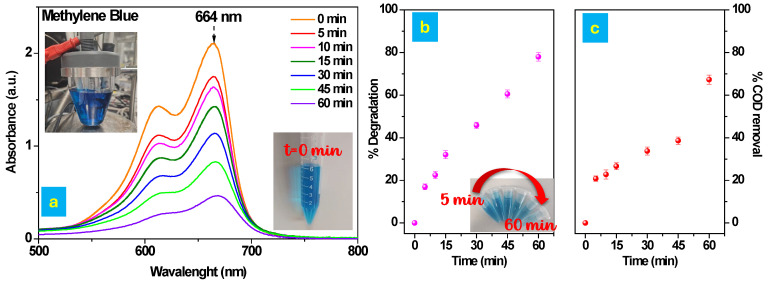
(**a**) Variation in the absorbance of MB during its electrochemical degradation on the Cu@AC-3DPE anode in 0.05 M NaCl aqueous solution containing 50 mg/L of MB, at a constant applied potential of 2 V and pH 6. The inset shows the electrochemical setup and the appearance of the MB solution at time t = 0 min. (**b**) Electrochemical degradation of MB (50 mg/L) on the Cu@AC-3DPE anode as a function of time, conducted at 2 V in the presence of 0.05 M NaCl as supporting electrolyte and pH 6. (**c**) Chemical Oxygen Demand (COD) removal (%) of MB (50 mg/L) on the Cu@AC-3DPE anode as a function of time, recorded at 2 V in 0.05 M NaCl electrolyte solution and pH 6.

The electrochemical degradation of MB in an aqueous solution (50 mg/L in 0.05 M NaCl) was investigated over a 60 min period, with the pH maintained at 6 and a constant potential of 2 V applied (Figure 5a). The electrochemical setup employed a platinum counter electrode and a Ag/AgCl reference electrode, as illustrated in Figure 1a. To monitor the progress of the degradation process, UV-Vis spectrophotometry was used, enabling the analysis of the discoloration over time. The absorption spectra of MB were recorded over time to monitor the progress of the degradation process. As shown in Figure 5a (orange profile), the spectrum acquired at time zero exhibits two characteristic bands in the visible region, located at approximately 612 nm and 664 nm. These bands correspond to the dimer (MB^+^)_2_ and the monomer MB^+^, respectively [39,58,59]. The absorption peak at 664 nm, being the most intense, was used to estimate the dye concentration throughout the experiment. Both spectral bands progressively decreased during electrolysis, indicating a continuous decline in MB concentration. Figure 5b shows the time-dependent degradation profile of MB. Additionally, a gradual loss of the blue coloration of the solution was visually observed, as depicted in Figure 5b. Moreover, COD analysis was performed over time (see Figure 5c). The figure shows a progressive increase in COD removal, reaching approximately 68% mineralization of methylene blue after 1 h of treatment.

##### Effect of NaCl Concentration

**Figure 6:**
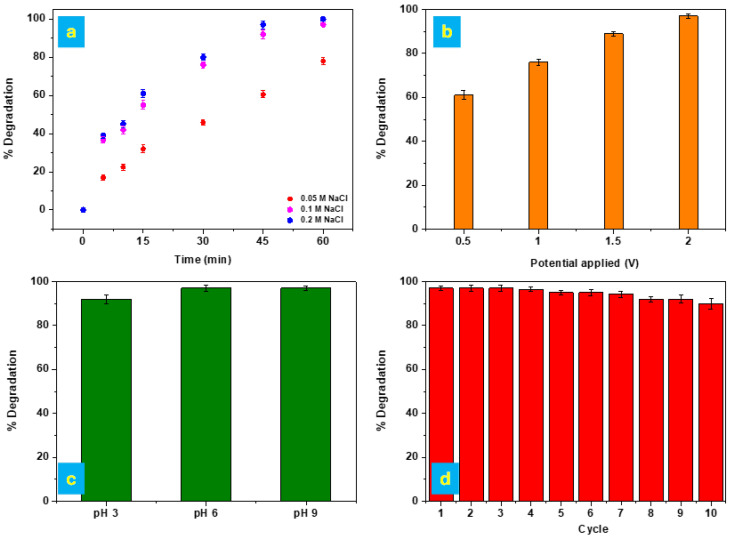
(**a**) Electrochemical degradation of methylene blue (MB, 50 mg/L) on the Cu@AC-3DPE anode as a function of time at varying NaCl concentrations, under a constant applied potential of 2 V and pH 6. (**b**) Electrochemical degradation efficiency of MB (50 mg/L) on the Cu@AC-3DPE anode after 60 min at different applied potentials, in the presence of 0.1 M NaCl and pH 6. (**c**) Effect of initial solution pH on the electrochemical degradation of MB (50 mg/L) after 60 min, using the Cu@AC-3DPE anode at 2 V and in 0.1 M NaCl. (**d**) Reusability performance of the Cu@AC-3DPE anode over successive cycles for the electrochemical degradation of MB (50 mg/L) under optimal operational conditions.

Figure 6a shows the effect of salinity on the degradation of MB. Experiments were conducted at different NaCl concentrations, ranging from 0.05 M to 0.2 M. As shown in Figure 6a, the degradation of MB increased significantly as the NaCl concentration rose from 0.05 M to 0.1 M, with degradation rates exceeding 80% after 45 min of treatment. The electrochemical oxidation of chloride ions at the anode surface generated active chlorine species (Cl_2_, HClO, and ClO^−^) [60], which are well known for their strong oxidative potential toward organic compounds [61]. However, further increasing the NaCl concentration from 0.1 M to 0.2 M did not result in a significant enhancement of the degradation rate. This is likely due to the excessive presence of NaCl hindering the mass transfer of pollutants to the anode surface and reducing the effective removal of contaminants by reactive free radicals [62]. Consequently, a NaCl concentration of 0.1 M was considered optimal for subsequent experiments.

##### Effect of Applied Potential

Figure 6b shows the removal of MB after 60 min as a function of the applied voltage. The figure illustrates that the removal efficiency increases with increasing voltage, reaching 61.15%, 76.36%, 89.1%, and 97.03% at 0.5 V, 1.0 V, 1.5 V, and 2.0 V, respectively. This is because the applied voltage serves as the driving force for the electrochemical reaction and influences the polarization of the particle electrode. The degree of polarization increases with higher voltage, thereby enhancing electrochemical activity. Specifically, increasing the voltage from 1.5 V to 2.0 V results in an improvement in removal efficiency from 89.1% to 97.03%, corresponding to an approximate 8.2% increase. Consequently, an applied voltage of 2.0 V was considered optimal for subsequent experiments.

##### Effect of pH

It is well established that the pH of the solution plays a critical role in influencing the electrocatalytic oxidation activity of the anode material [62]. In the present study, the MB removal efficiency remained above 90% across all tested pH values after 60 min of electrochemical treatment, demonstrating the system’s broad applicability under different pH conditions (see Figure 6c). However, it was observed that mildly basic to near-neutral conditions were more favorable for the degradation process. In particular, the MB removal efficiency at pH 6 was comparable to that observed at pH 9, suggesting that the system performs optimally within this pH range. This behavior may be attributed to the enhanced stability of reactive oxygen species and improved interaction between dye molecules and the electrode surface under these conditions. These findings highlight the versatility and effectiveness of the electrode across a wide pH spectrum, with a slight preference for neutral to alkaline environments.

##### Reusability

**Figure 7:**
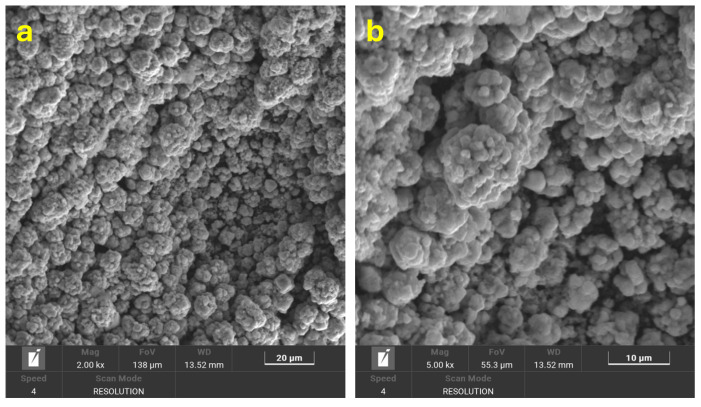
SEM images at different magnification of Cu@AC-3DPE anode after 10 cycles for the electrochemical degradation of MB (50 mg/L) under optimal operational conditions (**a**,**b**).

To evaluate the reusability of the electrode, a cyclic degradation test was conducted. Following the 97% removal of MB within 1 h, a fresh solution containing 50 mg/L of MB was introduced into the system using the same electrode. The dye was successfully degraded again within the same time frame. This procedure was repeated for a total of 10 consecutive cycles, consistently achieving nearly 97% removal efficiency in each cycle (see Figure 6d). These findings confirm the stability, durability, and excellent reusability of the electrode, which maintained high degradation performance throughout all ten cycles without any noticeable loss in efficiency. The stability observed during the tests also reflects the robustness of the polymer matrix, which maintained its structural integrity despite activation treatments and repeated electrochemical cycles. Figure 7 reports the SEM images of the Cu@AC-3DPE anode after 10 reuse cycles. The images clearly show that the electrode did not undergo any morphological modification, confirming its stability under the applied operating conditions.

#### 3.3.2. Degradation in Real Wastewater

**Figure 8:**
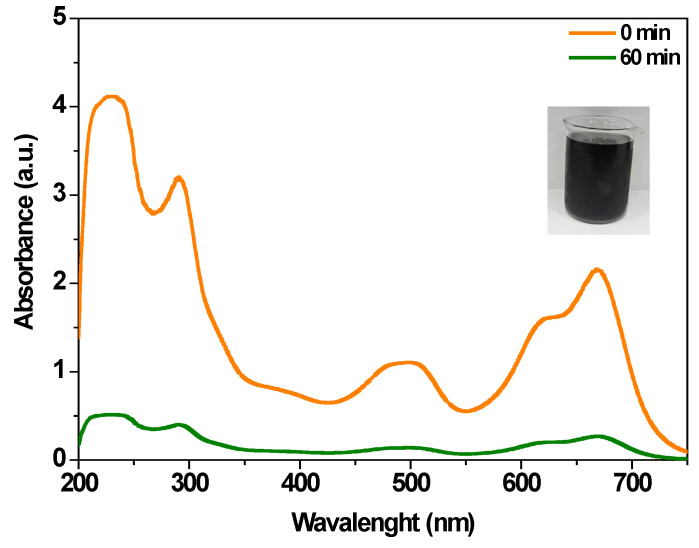
UV–Vis spectra of real wastewater in the presence of 50 mg/L MB, MO, SY, MR, and 10 mg/L of phenol before and after electrochemical oxidation using Cu@AC-3DPE anode under optimal operating conditions.

In most studies, electrochemical processes are evaluated using only a single dye or a few structurally related compounds, which limits their practical applicability to complex wastewater. Therefore, it is essential to develop systems based on electrodes that are inexpensive, durable, and easy to fabricate, capable of operating continuously and treating matrices that better represent real environmental conditions. In this context, the Cu@AC-3DPE anode was tested under real wastewater conditions. Since the availability of open industrial discharge sites in the Salerno area is limited, water samples were collected from a local channel primarily containing mixed domestic wastewater. The physicochemical characteristics of the samples were consistent with typical municipal wastewater, with average values of COD around 200 mg/L and BOD_5_ around 100 mg/L. The natural concentration of MB in the samples was negligible; therefore, to better simulate industrially contaminated effluents, the wastewater was spiked with approximately 50 mg/L of each dye (methyl red, MR; sunset yellow, SY; methyl orange, MO; and methylene blue, MB) and 10 mg/L of phenol. After one hour of treatment (Figure 8), MB removal reached ~93%, while significant degradation was also achieved for the main pollutants, with removal efficiencies of 86% for MR, 89% for SY, 88% for MO, and 95% for phenol.

#### 3.3.3. Effect of 3D-Printed Electrode Geometry

**Figure 9:**
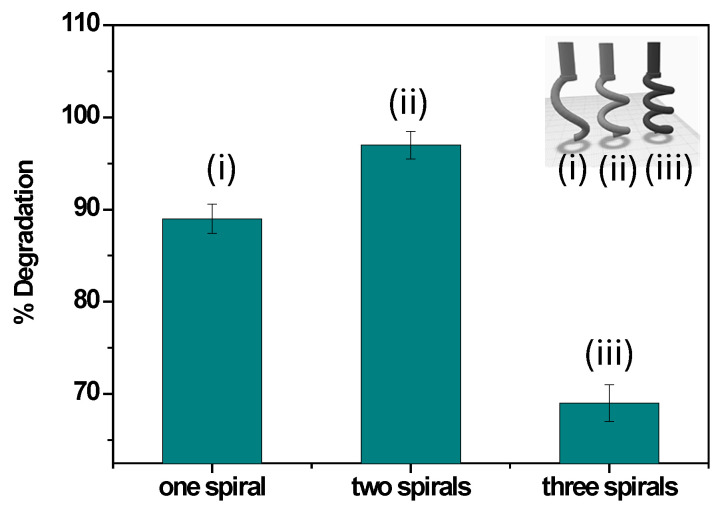
Electrochemical degradation of methylene blue (MB, 50 mg/L) using the Cu@AC-3DPE anode with a different geometry under optimal operating conditions.

A systematic investigation was carried out to assess the effect of electrode geometry on the electrochemical degradation of methylene blue (MB) (see Figure 9). Three helical configurations comprising one, two, and three spirals were fabricated and compared under identical operating conditions in NaCl electrolyte to evaluate if electrode geometry could enhance contact between the electrode and the electrolyte, facilitate vertical flow dynamics, and promote rapid detachment of gas bubbles from the surface, critical factors to prevent surface passivation and to maintain high catalytic efficiency [45]. The total length of each electrode was 5 cm, consisting of one, two, or three spirals, respectively. The optimized FDM printing parameters were those reported in Section 2.2. In terms of performance, the single-spiral electrode ensured good contact with the electrolyte but provided a limited reactive surface area, achieving approximately 89% MB removal. The three-spiral electrode increased the available surface area; however, its more complex geometry restricted solution circulation and promoted the formation of inactive zones, reducing the efficiency to about 69%. In contrast, the two-spiral electrode represented the most effective compromise, combining sufficient active surface area with improved flow dynamics, achieving up to 97% MB removal. This configuration proved to be the most stable and efficient for the electrochemical degradation of dyes.

### 3.4. Mechanism

The electrochemical degradation of MB in chloride-containing electrolytes proceeds via both direct and indirect oxidation pathways. According to the literature, the predominant mechanism involves the in situ generation of reactive oxidizing species at the anode surface [63,64,65,66], though partial direct oxidation of MB on the electrode surface has also been reported [67]. Initially, water molecules are electrochemically discharged at the anode, producing hydroxyl radicals (OH), which are physisorbed onto the surface of the Cu@AC-3DPE electrode. Simultaneously, chloride ions (Cl^−^) in the electrolyte are oxidized through the following reactions:

2Cl^−^ → Cl_2_ + 2e^−^ (4)

Cl_2_ + H_2_O → HOCl + H^+^ + Cl^−^ (5)

HOCl ⇌ H^+^ + OCl^−^ (6)

The hypochlorous species (HOCl/OCl^−^), known for their strong oxidative potential, combine with adsorbed •OH radicals to form active intermediates such as Cu NPs on a modified electrode (HOCl). These reactive complexes attack MB molecules, breaking them down into simpler organic compounds and ultimately mineralizing them to CO_2_ and water. At the end of the cycle, chloride ions are regenerated in the bulk solution, allowing the process to continue efficiently.

**Figure 10:**
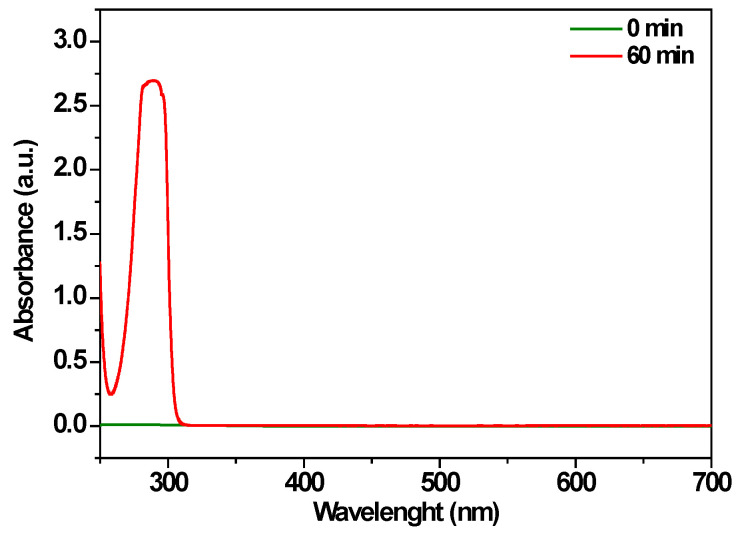
UV–Vis spectra of blank 0.1 M NaCl at pH 6 before and after electrochemical oxidation measurement at 2 V on the Cu@AC-3DPE electrode.

The control experiment, carried out in the absence of MB, confirmed the formation of hypochlorite anions from chloride-containing supporting electrolytes during electrolysis, as shown in Figure 10. The UV–Vis spectrum exhibits a peak at λ_max_ 290 nm after 60 min, which is characteristic of ClO^−^/HOCl species [68].

### 3.5. Polymer-Based Electrodes for Pollutant Degradation

Table 3 summarizes selected examples of polymer-based electrodes employed for the electrochemical degradation of organic pollutants [69,70,71]. Although these systems have demonstrated good performance, they often require prolonged treatment times or relatively harsh operating conditions. In contrast, the use of three-dimensional (3D) polymer electrodes in this field remains scarcely explored. Within this framework, the results obtained with the Cu@AC-3DPE anode are particularly significant: the electrode achieved approximately 97% removal of methylene blue within only 60 min, under mild operating conditions. This highlights not only the high efficiency of the system but also its innovative character compared to traditional approaches reported in the literature.

## 4. Conclusions

This work demonstrated the effectiveness of a novel 3D-printed anode, based on conductive PLA/CB and functionalized with copper nanoparticles, for the electrochemical removal of methylene blue from aqueous solution. The helical geometry of the electrode provided a large active surface area and efficient electrolyte contact. Importantly, the comparison of single-, double-, and triple-spiral configurations revealed that the double-spiral geometry achieved the best compromise between active surface and flow dynamics, leading to superior degradation performance. Optimization of operational parameters (voltage, pH, NaCl concentration) further enhanced the process, enabling high degradation efficiency (>97%) and significant COD reduction. The electrode also showed excellent stability over ten reuse cycles, confirming its durability and practical applicability. Beyond geometry and metallic decoration, a central outcome of this study is the demonstration that PLA/CB, a biodegradable polymer composite typically considered a prototyping filament, can act as the basis for high-performance electrochemical electrodes once properly activated and functionalized. This represents a significant step forward in rethinking the role of 3D-printing polymers, transitioning them from structural to functional applications. By combining sustainability, accessibility, and performance, PLA/CB-based electrodes can pave the way for new generations of cost-effective devices for water purification, sensing, and energy applications. Compared with other papers using metal-decorated 3D-printed electrodes, usually focused on analytical or energy-related fields, our approach uniquely highlights the synergistic role of electrode geometry, Cu NP functionalization, and polymer matrix activation in dye degradation. This integration establishes polymer-based 3D-printed electrodes as a promising, versatile, and scalable technology for electrochemical treatment of wastewater.

## Figures and Tables

**Table 1:** Metal-coated 3D-printed electrodes and their applications.

3D-Printed Electrode	Metal Coating	Application	Ref.
PLA/Carbon composite	Au electroplating	Heavy metal sensing (Hg^2+^)	[28]
Graphene/PLA	Ni microparticles (activated)	Glucose sensing	[29]
Graphene/PLA	Cu/Ni coating	Glucose and sucrose sensing	[30]
Graphene/PLA	Cu electroplating	CO_2_ reduction	[31]
Graphene/PLA	Ni-Pt coating	Hydrogen Evolution Reaction (HER)	[32]
Graphene/PLA	Ni-Co coating		[33]
PLA	Ni/Cu electrodeposition		[34]
Graphene/PLA	MoSₓ deposition (metal sulfide)		[35]
Ti-based 3DPE	MoS_2_ via ALD		[36]
Nano-carbon (3DPE)	MXene + dichalcogenides (MoS_2_, WS_2_, WSe_2_)		[37]

**Table 2:** Material properties.

Property	Value/Description
Base material	PLA
Characteristics	Low odor, non-toxic, renewably sourced
Molecular structure	Amorphous
Additives	Carbon black/Polymer blend
Density	~1.24 g/cm^3^
Minimum bend diameter	25 mm (Ø 1.75 mm)
Melting point (Tm) onset	~155 °C (310 °F)

**Table 3:** Comparison of electrochemical degradation performance with the literature.

Materials	Conditions	Pollutants	Removal Efficiency@Time	Ref.
Cu@AC-3DPE	pH: 6.0, V: 2 V, C(MB): 50 mg/L, C(NaCl): 0.1 M	MB	≈97%@60 min	This Work
Carbon–PTFE diffusion	pH = 3.0, Applied current = 33.3 mA/cm^2^, C(Ponceau 4R) = 254.0 mg/dm^3^, V = 130.0 cm^3^, C(Na_2_SO_4_): = 0.05 M	Ponceau 4R	100%@6 h	[69]
PANI/Gr	pH = 3.0, V: −0.6V, C(MO) = 50 mg L^−1^, C(Na_2_SO_4_): = 0.05 M	MO	100%@90 min	[70]
Ti/PbO_2_-Cr-PEDOT	pH: n.d. Applied current = 15 mA/cm^2^, C(Phenol) = 1000 mg/L, C(Na_2_SO_4_): = 0.028 M	Phenol	100%@120 min	[71]

PTFE → *Polytetrafluoroethylene*; PANI → *Polyaniline*; MO → *Methyl orange*; and PEDOT → *Poly(3,4-ethylenedioxythiophene)*.

## Data Availability

The original contributions presented in this study are included in the article material. Further inquiries can be directed to the corresponding author.
